# P-1696. Antibiotic consumption and resistance among admitted patients with urinary tract infection in the Philippines: Results from a longitudinal Global Point Prevalence Survey

**DOI:** 10.1093/ofid/ofae631.1862

**Published:** 2025-01-29

**Authors:** Mari Rose Aplasca De los Reyes, Jemelyn Garcia, Ines Pauwels, Ann Versporten, Rhenalyn V Bo, Erika Vlieghe

**Affiliations:** Research Institute for Tropical Medicine, Muntinlupa City, National Capital Region, Philippines; Research Institute for Tropical Medicine, Muntinlupa City, National Capital Region, Philippines; University of Antwerp, Antwerp, Antwerpen, Belgium, Wilrijk, Antwerpen, Belgium; University of Antwerp, Antwerp, Antwerpen, Belgium, Wilrijk, Antwerpen, Belgium; Research Institute for Tropical Medicine, Muntinlupa City, National Capital Region, Philippines; UZA, Antwerp, Antwerpen, Belgium

## Abstract

**Background:**

Urinary Tract Infection (UTI) is a common diagnosis among hospitalized patients for which *Escherichia coli* (*E.coli*) is the most common etiologic agent. Recently, increasing antimicrobial resistance of *E. coli* to common antibiotics has been reported in the Philippines. We describe the prevalence of antimicrobial use for UTI in Philippine hospitals from 2017-2022 and compare this with the antimicrobial resistance pattern of urinary *E. coli* generated by the Department of Health, Antimicrobial Resistance Surveillance Program.

**Figure:**
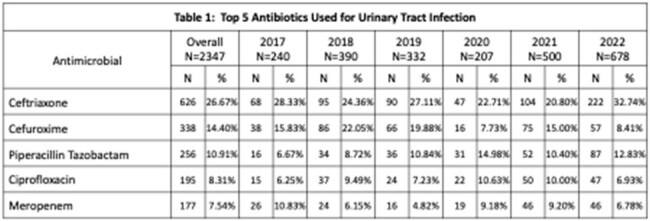

**Methods:**

Since 2017, the Philippines has been conducting point prevalence surveys (PPS) on antimicrobial consumption using the standardized and validated methodology of the Global-PPS. All admitted patients on antimicrobials at 8 AM on the day of the survey were included. Type of infections and their corresponding antimicrobial prescriptions were recorded.

**Figure:**
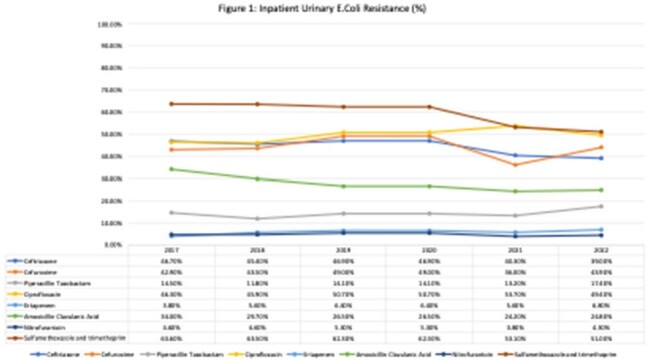

**Results:**

Data were collected in 62 hospitals in the Philippines. Overall, three percent (N= 2242; 3.3%) of patients admitted in Philippine hospitals on the day of the PPS were treated with antimicrobials for UTI (lower UTI: 1,8%, upper UTI:1.5%). Ceftriaxone, cefuroxime, piperacillin-tazobactam, ciprofloxacin and meropenem were the top five antibiotics used (table 1), all of which are classified under the WATCH list of the WHO AWaRe classification. Seventy-seven percent of all antibiotics were prescribed empirically. Based on the antimicrobial surveillance data of the Department of Health, the resistance rates of *E. coli* to these antibiotics were high with on average more than 40% resistance to cefuroxime, ceftriaxone and ciprofloxacin (figure 1).

**Conclusion:**

There is an opportunity to strengthen hospital antimicrobial stewardship programs, with particular emphasis on pre-authorization and audit and feedback strategies. Consideration may be given to placing ceftriaxone, cefuroxime, and ciprofloxacin under restricted categories requiring approval by the AMS clinician prior to empiric use. Importantly, local antibiograms should be reviewed on a regular basis and should be used as a basis for empiric antimicrobial recommendations in hospitals. Repeated point prevalence surveys are needed to measure the impact of interventions implemented by hospital AMS committees.

**Disclosures:**

**All Authors**: No reported disclosures

